# The Effect of Exercise Intensity on Total PYY and GLP-1 in Healthy Females: A Pilot Study

**DOI:** 10.1155/2017/4823102

**Published:** 2017-02-13

**Authors:** Jillian R. Hallworth, Jennifer L. Copeland, Jon Doan, Tom J. Hazell

**Affiliations:** ^1^Department of Kinesiology and Physical Education, University of Lethbridge, Lethbridge, AB, Canada T1K 3M4; ^2^Department of Kinesiology and Physical Education, Wilfrid Laurier University, Waterloo, ON, Canada N2L 3C5

## Abstract

We compared the acute response of anorexigenic signals (total PYY and GLP-1) in response to submaximal and supramaximal exercise. Nine females completed three sessions: (1) moderate-intensity continuous training (MICT; 30 min; 65%  VO_2max_); (2) sprint interval training (SIT; 6 × 30 sec “all-out” cycling sprints with 4 min recovery); or (3) control (CTRL; no exercise). PYY and GLP-1 were measured via blood samples drawn before, immediately after, and 90 min after exercise. Perceptions of hunger were rated using a visual analogue scale at all blood sampling time points. There was a session × time interaction for GLP-1 (*p* = 0.004) where SIT and MICT (*p* < 0.015 and *p* < 0.001) were higher compared to CTRL both immediately and 90 min after exercise. There was a main effect of time for PYY where 90 min after exercise it was decreased versus before and immediately after exercise. There was a session × time interaction for hunger with lower ratings following SIT versus MICT (*p* = 0.027) and CTRL (*p* = 0.031) 90 min after exercise. These results suggest that though GLP-1 is elevated after exercise in women, it is not affected by exercise intensity though hunger was lower 90 min after exercise with SIT. As the sample size is small further study is needed to confirm these findings.

## 1. Introduction

In order to develop evidence-based guidelines for weight management, it is important to understand how exercise affects energy balance (energy intake-energy expenditure). While increasing energy expenditure through exercise seems like a viable strategy to generate an energy deficit, its effectiveness on subsequent weight loss in the absence of dietary restriction is often less than desired [1, 2] and though many exercise interventions improve body composition in males, they are less effective in females [2–4]. This may be a result of exercise causing a compensatory increase in energy intake, preventing the energy deficit required for an improvement in body composition [5] as females may partially compensate (~30%) for varying levels of exercise-induced energy expenditure by increasing energy intake [4]. Exercise protocols that can increase energy expenditure without increasing energy intake could be a viable way to develop and improve weight management guidelines.

One such exercise protocol may be sprint interval training (SIT), which is an intense form of high-intensity interval training (HIIT) involving brief 30 sec “all-out” efforts followed by 4 min of active rest [6, 7] and has demonstrated similar physiological adaptations to traditional moderate-intensity (50–75%  VO_2max_) continuous training (MICT) completed for much longer duration (30–60 min). Despite its vastly reduced training volume [8], SIT has many health benefits [9] including potential reductions in body fat [8, 10]. While these fat mass losses may be attributed to a protracted increase in postexercise metabolism [8, 11, 12] or an increase in fat utilization [11, 13], others have questioned whether these mechanisms could fully explain the magnitude of fat loss [14]. Therefore, alternative mechanisms need to be explored such as potential exercise-induced effects on appetite-regulating hormones [15, 16] and subsequent energy intake [17].

At present, little is known about the acute effect of exercise on appetite-regulating hormones and the resulting perception of hunger in women. The regulation of energy intake is a complex pathway that includes meal initiation, meal termination, meal frequency, and nutrient intake as well as long-term regulation of energy intake in relation to body energy requirement [18]. Each of these components are experienced and acted upon as a result of the integration of various physiological and psychological factors, including hormonal signaling. Important anorexigenic signals, peptide tyrosine-tyrosine (PYY) and glucagon-like peptide-1 (GLP-1), are mainly involved in the potential satiating effect of exercise [15, 16]. A change in these hormones stimulates a cascade of events eventually terminating at neurotransmitter receptors in the hypothalamus, affecting appetite and subsequent energy intake. Though several studies have found increases in GLP-1 and PYY with MICT in males [19–22], only a few have examined the responses of these hormones to SIT [13, 23]. In females, concentrations of PYY and GLP-1 have been shown to increase following MICT [24–26], though no effect of exercise intensity was shown at submaximal intensities in highly trained females [25]. However, to date, no study has examined the response of anorexigenic signals to supramaximal SIT in females. While exercise is an effective method of increasing energy expenditure and is often encouraged for weight management, its efficacy in appetite suppression and weight loss may vary with exercise intensity.

Considering that exercise may influence appetite-regulating hormones through several mechanisms, many of which may be intensity-dependent [15], comparing the hormonal response to exercise at different intensities (submaximal and supramaximal) in females is necessary. Thus, the aim of the present study was to compare the total PYY, GLP-1, and hunger response between a traditional moderate-intensity aerobic exercise session and a SIT exercise session in females. We hypothesized that females would have an increased PYY and GLP-1 response to SIT due to the more intense stimulus [15].

## 2. Materials and Methods

### 2.1. Participants

Thirteen healthy, active females volunteered and provided written informed consent to participate. Four participants withdrew from the study, specifically, two due to lack of time, one who moved cities, and one where there were problems with obtaining two blood draws. Therefore, nine participants were available for analysis. The data presented here are part of a larger research project assessing the role of exercise intensity on appetite-regulating hormones. The male data have been previously published in Appetite [27]. All participants were recreationally active which was classified as participating in moderate-intensity exercise for a minimum of 30 min, at least three times per week. None of the participants were smokers or currently taking prescribed medication (aside from hormonal contraceptives including combination pill, progesterone only pill, and intrauterine device), and all were screened for history of diabetes, eating disorder, drug or alcohol abuse, coronary heart disease, food allergies, and medication use (specifically for those known to affect appetite or hypertension or induce weight loss). To control for phase of menstrual cycle, all participants, including those on oral contraceptives, completed all sessions during the early follicular phase (days 1–10) of the menstrual cycle, based on self-reported onset of menstruation. All were instructed to perform no physical activity or ingest any caffeine for 48 h prior to any laboratory visit. The University of Lethbridge Human Subject Research Committee approved all experimental procedures in accordance with the ethical standards of the 1964 Declaration of Helsinki.

### 2.2. Familiarization Session

Prior to the experimental sessions, each participant attended a familiarization session in which they were screened for exclusion criteria and introduced to the study protocol and equipment. Participants were acclimatized to the exercise equipment as well as the types of effort required during the different exercise protocols. Height and weight were measured using a mechanical beam scale (Health-o-meter Professional, Sunbeam Products, Inc., Illinois, USA). Body mass index (BMI) was calculated as body mass (kg) over height squared (m^2^). Body density was calculated from skinfold measures obtained using a seven-site formula [28] and the Siri equation to determine body fat percentage. Participants then completed a maximal oxygen uptake (VO_2max_) with the gas exchange measured directly using an online breath-by-breath gas collection system (Quark CPET, Cosmed, Chicago, Illinois, USA). The test followed a graded protocol to exhaustion on a mechanically braked cycle ergometer (Model 874-E, Monark Exercise, Stockholm, Sweden). Following a 5 min warm-up at 70 rpm and 1 kg resistance, participants maintained a 70 rpm cadence with 0.5 kg resistance added every 2 min until volitional fatigue was reached or 70 rpm could no longer be maintained. Heart rate (HR) was measured throughout the test using a Polar HR monitor (FT7, Polar Electro Oy, New York, USA) and ratings of perceived exertion (RPE) were assessed simultaneously with each increase of resistance throughout the duration of the test [29]. Verbal encouragement was provided throughout the test. At the end of the test, VO_2max_ (greatest 30 sec average) was established by the presence of a plateau in VO2 values (<1.35 mL·kg^−1^ increase in VO_2_) or when two of the following criteria were obtained: (1) a respiratory exchange ratio (RER) value >1.15, (2) HR within ±10 bpm of age predicted maximum HR (220-age), and/or (3) visible subject exhaustion [30]. Upon determination of VO_2max_, 65% of this value was calculated as the target exercise intensity during the MICT session.

### 2.3. Experimental Sessions

**Figure 1:**
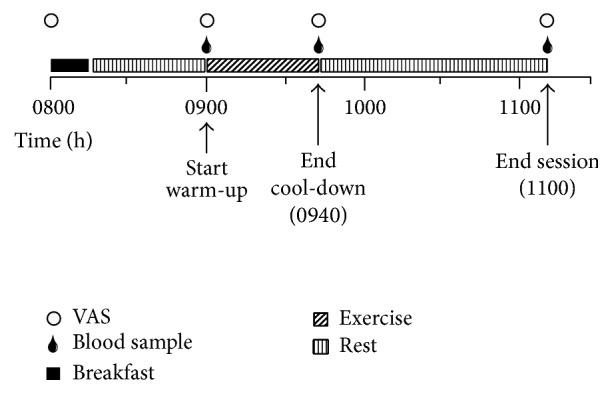
Overview of experimental session. VAS: visual analogue scale.

At least one week after the familiarization session, participants completed a randomized three-way crossover study with all experimental sessions (Figure 1) performed at least 1 week apart. The three experimental sessions consisted of 30 minutes of (1) moderate-intensity continuous training (MICT; 65%  VO_2max_); (2) sprint interval training (SIT); and (3) no exercise control (CTRL). For 24 h prior to the first experimental session, participants completed a 24 h dietary journal where they recorded their food intake, including the quantity of each food and beverage consumed for each meal. Participants were asked to replicate this same diet for the 24 h prior to each subsequent experimental session. On the morning of each session, participants arrived at the laboratory at 0800 h in a fasting state (no food or drink except water for a minimum of 10 h) and remained in the laboratory for the next ~3 h. Upon arrival for each session participants randomly drew a slip of paper to determine what session they would be doing that day (SIT, MICT, or CTRL). Participants consumed a standardized breakfast (4 kcal·kg^−1^; 16.7 kJ·kg^−1^) consisting of an energy bar (up to 1050 kJ; Clif Bar & Company, California, USA), plain Quaker rice cake (up to an additional 147 kJ; PepsiCo, Ontario, Canada), and natural peanut butter (up to remaining allotted kJ; Costco Wholesale, Washington, USA). Participants were provided 15 min to consume breakfast. Water was provided ad libitum throughout experimental sessions. After allowing sufficient time for digestion (~45 min), the exercise began at 0900 h and included a 5 min standardized warm-up, a 30 min exercise protocol (27 min for SIT with an additional 3 min rest prior to warm-up to match), and a 5 min cool-down. Upon completion of exercise (0940 h), participants remained in the laboratory for an additional 90 min and rested quietly (reading or using a laptop). Venous blood samples were obtained (detailed below) at 0900 h (before exercise), 0940 h (immediately after exercise), and 1110 h (90 min after exercise). Perceptions of hunger were assessed before breakfast and at each blood sampling time-point [31, 32]. Participants were asked to indicate their current level of hunger (“How hungry do you feel?”) on a visual analogue scale (VAS) that was anchored at each end with contrasting statements (i.e., “I am not hungry at all” and “I have never been more hungry”). A hedonic scale was not used in the current study as with a crossover design the hedonic levels would be matched across conditions. Identical procedures were followed during the CTRL session with the exception of the exercise period (0900–0940 h) during which participants rested quietly (i.e., reading or using a laptop).

### 2.4. Exercise Protocols

Both exercise sessions were performed on a cycle ergometer (model 874-E, Monark Exercise, Stockholm, Sweden) and heart rate was recorded continuously using a HR monitor (as described above). The MICT session consisted of 30 min of continuous cycling at 65%  VO_2max_ where work rate was determined individually using the American College of Sports Medicine's metabolic equation for gross VO_2_ during leg cycling, specifically VO_2_ = (work rate (watts)(body mass (kg)^−1^)) · 10.8 + 7. Gas exchange was measured for 1 min every 5th minute of exercise using an online breath-by breath gas collection system (described above) and work rate modifications were made accordingly. The SIT protocol was structured as six 30 s “all-out” efforts against 10% of body mass, separated by 4 min recovery periods. Oxygen consumption was not measured during SIT, though we have measured this previously [33, 34]. Instructions to begin pedaling as fast as possible against the inertial resistance of the ergometer were given prior to each bout and the appropriate load was applied instantaneously (within 3 sec). Verbal encouragement was provided throughout.

### 2.5. Blood Processing

All blood samples were collected via venipuncture from the antecubital vein while participants were in a seated position. Venous blood samples were collected into prechilled 6 mL EDTA tubes (Becton Dickinson Vacutainer K2 EDTA [lavender top] tubes, New York, USA). A protease inhibitor cocktail (BioTool, Ontario, Canada) was added to a final 1x concentration to prevent hormone degradation. Samples were centrifuged at 1780 rpm for 10 min at 4°C and supernatants were aliquoted, stored at −80°C, and batch analyzed at the end of the study to reduce inter- and intra-assay variability. The concentration of PYY in the plasma samples was determined using Millipore human PYY total ELISA kits (EMD Millipore PYY Total ELISA Kit, Millipore Corporation, Billerica, MA, USA) and the concentration of GLP-1 was determined using Millipore GLP-1 total ELISA kits (EMD Millipore GLP-1 Total ELISA Kit, Millipore Corporation, Billerica, MA, USA) following the manufacturer's instructions. The sensitivity of the assays was 1.4 pg·mL^−1^ for PYY and 1.5 pmol·50 *μ*L^−1^ for GLP-1. All samples were assayed in duplicate and intra-assay variation was 5.8 ± 1.9% and 4.5 ± 2.3% for GLP-1 and PYY, respectively, and interassay variation was 8.0 ± 4.8% and 7.3 ± 3.6% for GLP-1 and PYY, respectively.

### 2.6. Statistical Analysis

All data were analyzed using the Statistical Package for Social Sciences (SPSS) software v22.0 for Windows (SPSS, Chicago, IL). Hormone concentrations (GLP-1 and PYY) were analyzed as absolute change in concentration from baseline using separate 3 × 3 repeated measures ANOVA (session × time). For significant main effects, a Bonferroni adjustment was used for multiple pairwise comparisons. With regard to the anorexigenic hormones, both absolute concentrations and relative changes from baseline resulted in similar statistical output; therefore subsequent presentation of data will focus on the change in hormone concentrations relative to baseline. For differences in perceptions of hunger, a 3 × 3 repeated measures ANOVA (session × time) was performed using the preexercise measure as baseline. This subjective data was also normalized to baseline values and analyzed as a change from baseline. A one-way repeated measures ANOVA was used to compare AUC values for both hormones. Effect sizes were calculated using Cohen's *d* to determine the magnitude of an effect independent of sample size. Small effect sizes are considered as *d* < 0.2, moderate effect sizes as *d* = 0.2–0.8, and large effects sizes as *d* > 0.8. Statistical significance was set at *p* < 0.05. All results are presented as mean ± standard deviation.

## 3. Results

### 3.1. Participants

Nine females completed all experimental sessions and participant characteristics are presented in Table 1.

### 3.2. Exercise Intensity

During the MICT session, VO_2_ reached steady state at ~65% of VO_2max_ (27.37 ± 3.0 mL·kg^−1^·min^−1^). The peak HR achieved was significantly higher during SIT (171.6 ± 8.1 bpm) versus MICT (153.1 ± 15.2 bpm, *p* = 0.003); however, average HR did not differ significantly between exercise sessions (MICT = 137.3 ± 14.8 bpm; SIT = 135.5 ± 13.9 bpm, *p* = 0.774).

### 3.3. Plasma GLP-1 Response to Exercise

**Figure 2:**
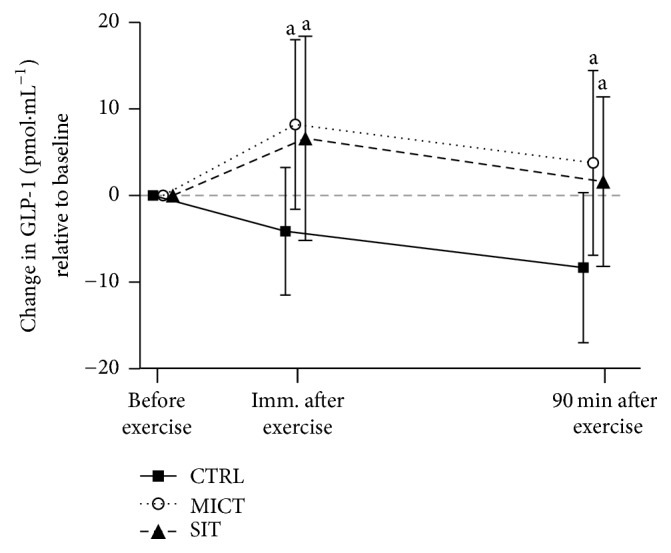
Changes in GLP-1 concentrations across all time points relative to baseline for each experimental session. CTRL: control (no exercise); MICT: moderate-intensity continuous training; SIT: sprint interval training.* Note*. ^a^Significantly different versus CTRL (*p* < 0.015).

Circulating concentrations of total GLP-1 during all three experimental sessions, presented as change from baseline, can be seen in Figure 2. There was a significant session by time interaction (*p* = 0.004) where MICT and SIT were increased versus CTRL (large effect sizes) at immediately (*p* < 0.001, *d* = −1.4; *p* = 0.002, *d* = −1.9) and 90 min after exercise (*p* < 0.001, *d* = −1.2; *p* = 0.015, *d* = −1.0), respectively.

### 3.4. Plasma PYY Response to Exercise

**Figure 3:**
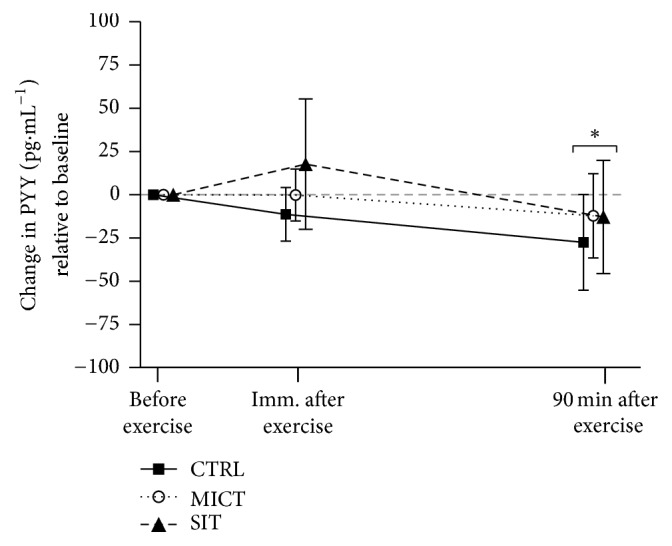
Changes in PYY concentrations across all time points relative to baseline for each experimental session. CTRL: control (no exercise); MICT: moderate-intensity continuous training; SIT: sprint interval training.* Note*. ^*∗*^Significantly different versus before exercise (*p* = 0.016) and immediately after exercise (*p* = 0.013).

Circulating concentrations of total PYY during all three experimental sessions, presented as change from baseline, can be seen in Figure 3. There was a main effect of time for PYY (*p* = 0.007), where 90 min after exercise concentrations were lower (moderate to large effect sizes) compared to before exercise (*p* = 0.016, *d* = −0.9) and immediately after exercise (*p* = 0.013, *d* = −0.3). There was no main effect for session (*p* = 0.249) and no session × time interaction (*p* = 0.256).

### 3.5. Hunger Ratings

**Figure 4:**
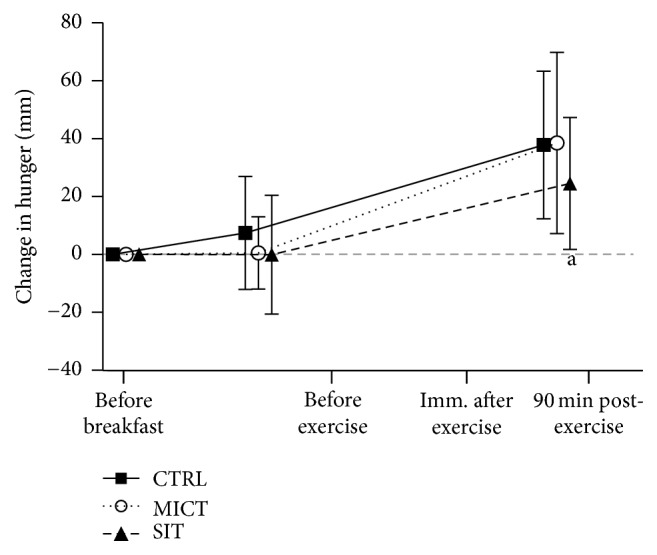
Perceptions of hunger at all time points during each experimental session. CTRL: control (no exercise); MICT: moderate-intensity continuous training; SIT: sprint interval training.* Note*. ^a^Significantly different versus CTRL and MICT (*p* < 0.027).

Perceptions of hunger during all three experimental sessions can be seen in Figure 4. There was a significant session × time interaction (*p* = 0.025) where SIT was decreased (moderate effect size) versus CTRL and MICT 90 min after exercise (*p* = 0.027, *d* = 0.06 and *p* = 0.031, *d* = 0.5, resp.). There were no other significant differences.

## 4. Discussion

To our knowledge, this is the first study to compare the total GLP-1 and PYY response to MICT and SIT among recreationally active females. Our results demonstrate that total GLP-1 increased in response to exercise compared to CTRL with no difference between MICT and SIT. Total PYY concentrations were lower 90 min after exercise compared to before and immediately after exercise with no difference between sessions. Feeding did not appear to influence the perception of hunger (perhaps the meal was too small), though both MICT and SIT resulted in lower ratings of hunger compared to CTRL after exercise where only SIT still had depressed perceptions of hunger 90 min after exercise.

The research on the effects of exercise on GLP-1 is not as prominent as other hormones such as PYY, leptin, and ghrelin. Previous studies generally demonstrate that MICT results in an increase in GLP-1 in both males and females [22, 26, 35]. However, several studies have demonstrated no effect of exercise [13, 25, 36] with no effect of increasing intensity during submaximal continuous exercise [25] and no effect of SIT [13]. Our current results demonstrate that postprandial exercise significantly increased total GLP-1 concentrations after exercise (immediately and 90 min) with no difference between MICT and SIT. To our knowledge, this is the first study in females to examine the GLP-1 response to both MICT and SIT and report a significant change in GLP-1 following SIT.

Previous studies have generally shown that MICT [13, 22, 23, 35–37] and SIT [13, 23, 36] increase PYY after exercise in males. There have been limited studies in females as only two have been completed to date demonstrating increase similar to males following MICT [24, 26], though to the authors' knowledge no study has measured the PYY response to SIT in females. We found no effect of exercise (MICT or SIT) on PYY in females as the present results illustrate no effect of session on total PYY, only an effect of time where PYY was lower at 90 min after exercise compared to before exercise. While our findings are not in agreement with the majority of previous literature there are several other reports of no effect of MICT [21, 38–40] or HIIT [37] on PYY and could involve our exercise being performed in the postprandial state while several other studies are in the fasting state [19, 21, 24, 36, 38, 41]. Some individuals perform exercise after consuming an energy drink or bar and so we believe it is important to examine exercise after eating as well as in a fasting state. It is also possible that differences between studies are explained by differing intensity or duration of exercise, depending on the mechanism by which exercise may influence PYY concentrations.

With no clear understanding of the mechanisms involved in the effect of exercise on appetite-regulation we have recently highlighted several possibilities [15]. Potential increases in sympathetic nervous system activity can influence both GLP-1 and PYY as catecholamines can stimulate the release of both hormones from intestinal L-cells [42, 43]. Another possibility involves the contraction-induced cytokine interleukin-6 (IL-6), with interesting evidence of its role in increasing GLP-1 in animals [44, 45], though no research has been conducted to date in humans. Elevated IL-6 concentrations have been shown to positively correlate with energy intake in humans [46], though whether this is mediated through GLP-1 remains to be determined. Future work should begin to focus on the potential mechanisms by which exercise alters appetite-regulation in an effort to unravel the complex findings in the literature.

Elevated concentrations of these hormones are often accompanied by decreased perceptions of hunger [19, 22, 47]. Here we found that both MICT and SIT decrease the perception of hunger immediately after exercise in females, consistent with the increases in GLP-1. However, the perception of hunger dissociates from GLP-1 as the lower hunger ratings were maintained at 90 min after exercise only after SIT, while GLP-1 was still similar between MICT and SIT. There are several possible explanations for this apparent dissociation; the first and simplest is that these hormones are only one factor that can influence appetite and other hormones should be considered such as ghrelin. Second, measuring the active forms of these hormones may have provided an answer more in line with the perception of hunger response. Third, future research could be warranted to see if SIT increases leptin as SIT is known to increase postexercise fat oxidation [11, 13]. Fourth, perhaps we should have measured the circulating neuropeptides involved in appetite-regulation such as agouti-related peptide (AgRP) and alpha-melanocyte-stimulating hormone [48].

This study provides insight, specifically in females, into appetite-regulating peptides, perceptions of hunger, and how they may vary between exercise intensities that are often prescribed for weight loss. The study does have several limitations. First the sample size is small and included only young, healthy individuals of reproductive age. Further study is needed to confirm these findings and to explore these effects among inactive, obese, or postmenopausal women. Also, we measured total concentrations of both GLP-1 and PYY rather than their bioactive form which may have limited our conclusions [49] though many studies suggest that total concentrations reflect changes in other forms [13, 22, 23, 26, 36, 41, 50, 51]. Furthermore, measuring acylated ghrelin concentrations may have provided additional insight, as this hormone may be more responsive to exercise intensity (Hazell et al., 2016; Schubert et al., 2014). Finally, while participants were instructed to consume the same foods in the 24 h prior to sessions and to refrain from strenuous exercise and alcohol in the hours leading up to the session, their compliance is not guaranteed.

## 5. Conclusion

This is the first study to compare the response of total GLP-1, PYY, and perceptions of hunger between MICT and SIT in females. The primary finding of this study is that GLP-1 increased after both MICT and SIT though there were no effects of exercise on PYY. Though the similar increases in GLP-1 are in line with the decreased hunger immediately after exercise after both MICT and SIT, only SIT was associated with prolonged suppression of hunger 90 min after exercise. Future studies should examine the potential mechanisms involved in the exercise-induced changes in GLP-1 and hunger. Additionally, future studies are needed to determine if the acute response of these hormones following exercise translates into altered EI, with potential implication to weight management strategies.

## Figures and Tables

**Table 1:** Participant characteristics.

Age (y)	30.5 ± 7.9
Height (cm)	1.75 ± 0.15
Weight (kg)	72.4 ± 2.0
BMI (kg·m^2^)	23.5 ± 2.8
Body fat (%)	22.8 ± 4.3
VO_2max_ (mL·kg^−1^·min^−1^)	40.7 ± 5.4
